# Polyphenols and extracts from *Zingiber roseum* (Roxb.) Roscoe leaf mitigate pain, inflammation and pyrexia by inhibiting cyclooxygenase-2: an *in vivo* and *in silico* studies

**DOI:** 10.3389/fphar.2024.1344123

**Published:** 2024-02-14

**Authors:** Shakhawat Ahmed, Khondoker Shahin Ahmed, Md. Naiemur Rahman, Hemayet Hossain, Aixia Han, Peiwu Geng, A. F. M. Shahid Ud Daula, Abdullah Al Mamun

**Affiliations:** ^1^ Department of Pharmacy, Noakhali Science and Technology University, Sonapur, Bangladesh; ^2^ Chemical Research Division, Bangladesh Council of Scientific and Industrial Research (BCSIR), Dhaka, Bangladesh; ^3^ Central Laboratory of The Sixth Affiliated Hospital of Wenzhou Medical University, Lishui People’s Hospital, Lishui, Zhejiang, China

**Keywords:** *Zingiber roseum*, polyphenols, analgesic activity, anti-inflammatory activity, antipyretic activity

## Abstract

*Zingiber roseum* (Roxb.) Roscoe*,* a perennial herb from the Zingiberaceae family, has a long history of traditional use in the treatment of several ailments including pain, inflammation, fever, cough, arthritis, skin diseases, and liver infections*.* This study sought to confirm the efficacy of *Zingiber roseum* (Roxb.) Roscoe leaves methanol extract (ZrlME) as reported in traditional usage by evaluating its analgesic, anti-inflammatory, and antipyretic capabilities. In addition, *in silico* molecular docking of the metabolites identified in ZrlME was studied to verify the experimental outcomes. ZrlME demonstrated strong dose-dependent analgesic efficacy against all analgesic tests. ZrlME (400 mg/kg) showed higher anti-inflammatory activity than the standard in the carrageenan-induced paw edema test model. A significant reduction of rectal temperature (3.97°F↓) was also recorded at the same dose of ZrLME after 24 h of treatment. Seven polyphenolic metabolites were identified and quantified by HPLC-DAD analysis, including 3, 4- dihydroxy benzoic acid, (-) epicatechin, rutin hydrate, p-coumaric acid, trans-ferulic acid, rosmarinic acid, and myricetin. Strong binding affinities (ranges from −5.8 to −8.5 Kcal/mol) between the aforesaid polyphenols and cyclooxygenase-2 were discovered. Moreover, molecular dynamics simulations (MDS) demonstrated that these polyphenols exhibit significant COX-2 inhibitory activity due to their high stability in the COX-2 active site. In computational prediction, the polyphenols were also found to be nontoxic, and a variety of biological activities, such as antioxidant, analgesic, anti-inflammatory, antipyretic, and hepatoprotective, were observed. The results of this study revealed that ZrlME possesses notable analgesic, anti-inflammatory, and antipyretic properties.

## 1 Introduction

The social and healthcare systems are heavily burdened by pain, fever, and inflammatory consequences, which have been known to harm people for generations. The frequency and incidence of pain-related diseases are increasing day by day, with 30% of adults worldwide experiencing pain and inflammatory diseases and 20% receiving a chronic illness diagnosis each year (Javed et al., 2020). They commonly manifest in a diverse array of pathological circumstances, encompassing wounds, infections, tissue injury, inflammation, and many diseased states. Pain is an unpleasant feeling brought on by sensory and tissue damage, and it profoundly impacts many aspects of human functioning (Puebla Díaz, 2005). In addition to sleeplessness, anxiety, weariness, decreased appetite, and even limb dysfunctions, pain can occur in any part of the body, including the head, stomach, limbs, muscles, and joints (Chou et al., 2016). Inflammation is a complex process that is brought on by a variety of factors, including mechanical injury, tissue ischemia, pathogenic agents, and toxic substances. It is characterized by tissue alterations that allow for the rapid migration of immune cells to the sites of inflammation (Chen et al., 2018; Gupta et al., 2018). Fever, or pyrexia, is a complicated immune-physiologic illness that is brought on by a series of biochemical responses to inflammatory or infectious stimuli in the body (Muhammad et al., 2019). Inflammatory and pathogenic diseases, such as IL-6, CRF, IL-1, chemokines, and especially PGE2, cause the production of many endogenous pyrogens (Roth et al., 2006). Prostaglandin E2 (PGE2) is a key eicosanoid component of the central nervous system during fever in animals (Gaetano et al., 2010). Conventional non-steroidal anti-inflammatory medicines (NSAIDs) suppress the synthesis of prostaglandins, which are the most significant inflammatory mediators, by non-specifically blocking the activity of both COX-1 and COX-2 enzymes (Botting, 2010). For fever, pain, and inflammation, commonly recommended NSAIDs and narcotic analgesics are those with efficacy data. However, NSAIDs are frequently used to treat a variety of inflammatory disorders, but their prolonged use is associated with a number of negative side effects, including liver and kidney damage, gastrointestinal ulcers and bleeding, and gastrointestinal hemorrhage (Kwiecień et al., 2015). In this context, natural medications made from numerous medicinal plants can be compelling substitutes.

Medicinal plants are alluring sources of biologically active substances that can protect people from a range of serious ailments. An essential category of secondary metabolites consists of polyphenols, which are significant for the countless potential health benefits they may provide. They are classified into several subgroups, including tannins, quinones, curcuminoids, flavonoids, phenolic acids, lignans, coumarins, and stilbenes. Various biological effects can be attributed to these phenolic chemicals, including anti-thrombotic, antibacterial, immunomodulatory, vasodilatory, hepatoprotective, anti-inflammatory, analgesic, antipyretic, and anti-arthritic effects (Benavente-García et al., 1997; Middleton et al., 2000; Manach et al., 2005a). However, due to their negligible side effects, plant-derived metabolites have caught the attention of researchers as a possible substitute therapy for arthritis, inflammation, fever and other neuropathic pain.

*Zingiber roseum* (Roxb.) Roscoe, often known as “Jangli Adrak,” is an upright, perennial herb in the Zingiberaceae family. Despite its rarity, this plant can be found all over the Himalayas (Babu, 1977). It features long leaves, a tuberous rhizome, an upright stem, red blooms, and white seeds. *Z. roseum* features a ligule that is 1.2–1.5 cm long, dull red spikes with white colour bottom, a long peduncle, red petals with white bases, a bump in the rhizomes, a complete labellum with a light yellow edge (Rahman and Yusuf, 2013). The plant is indigenous to Bangladesh and can be found in South China, Thailand, and Myanmar. *Z. roseum* is utilized by the Munchingputtu Mandal tribes of India, Visakhapatnam District, to heal wounds, ulcers, night blindness, piles, coughs, swelling throats, stomachaches, and cardiac infections. It has also been used for centuries to cure dyspepsia, skin problems, coughs, liver infections, and fevers (Mahawer et al., 2023; Rahman et al., 2023). The rhizomes of *Z. roseum* are used by traditional healers in Bangladesh to cure rheumatic diseases, asthma, and wounds (Al-Amin et al., 2019). The acute toxicity assessment of this plant’s rhizomes indicates that they are safe for human consumption (Amanat et al., 2023). Despite the extensive historical usage of *Z. roseum* in traditional medicine, there remains a significant lack of understanding regarding the majority of its pharmacological effects. The present investigation aimed to evaluate the *in vivo* analgesic, anti-inflammatory, and antipyretic properties of ZrlME, with the objective of substantiating its traditional medicinal application. The current study’s objective was also to identify and analyze phenolic metabolites in ZrlME using HPLC-DAD analysis.

The docking technique is now widely used as a common computational tool for discovering new active metabolites and their affinity to specific receptors (Parenti and Rastelli, 2012). Using molecular docking studies, we also tried to identify the potential mechanism behind the analgesic, anti-inflammatory, and antipyretic activities of identified polyphenols. For molecular docking investigations, the target enzyme cyclo-oxygenase-2 (COX-2) was selected. In order to inhibit the production of prostaglandins, which are linked to pain, fever, and inflammation, we must inhibit the activity of the COX-2 enzyme, which is produced during an inflammatory response and results in pain, swelling, and stiffness (Hawkey, 1999). Therefore, we tried to figure out how well the identified chemicals bound to the cyclo-oxygenase-2 (COX-2) enzyme. In addition, to validate the results of the experiments, absorption, distribution, metabolism, excretion, and toxicity (ADMET) profile of the discovered polyphenols of ZrlME were also investigated.

## 2 Materials and methods

### 2.1 Plant materials

After collection from the Sitakunda hill region in Chattagram, Bangladesh, the plant specimen was identified and verified by Professor Shaikh Bokhtear Uddin, a faculty member in the Department of Botany at Chittagong University. The herbarium at Chittagong University has a voucher specimen of this plant species (Accession number. SBU11064).

### 2.2 Extract preparation

The leaves of the plant were clipped, cleaned, and pulverized into a fine powder after being shade-dried at room temperature. A clean glass container with a flat bottom filled with 400 g of the powder of the dried leaves of the plant and 1,500 mL of methanol was added. The mixture was allowed to sit at room temperature for 15 days while being sometimes shaken and stirred. After filtering with Whatman filter paper No. 1, the solution was concentrated using a rotary evaporator. The resultant extracts were then vacuum dried and stored in sealed glass vials in a refrigerator.

### 2.3 HPLC-DAD analysis

Shimadzu LC- 20A system (Tokyo, Japan) was used to analyze the extract’s phenolic content. The instrument setup and solvent system were followed according to Talukder et al., 2022; Talukder et al., 2022). The following settings were used: injection volume of 20 L, 0.5 mL/min flow rate, 5%–25% solution A for the first 0.01–20 min, 25–40 min, 40–60 min, 60 to 35 min, 30%–5% minutes, and 5% minutes for the next 40–50 min. Based on the peak area, the concentrations of each metabolite were determined, and the values were represented in mg per 100 g dry extract.

### 2.4 Experimental animals

The Swiss-albino mice, weighing between 20 and 25 g, were obtained from the animal house located at Jahangirnagar University in Dhaka, Bangladesh. The mice were housed in plastic cages with ambient illumination that alternated between 12 h of darkness and 12 h of light. During the duration of the study, mice were given unrestricted access to food and drinks. In each of the conducted studies, there were four distinct groups, with each group including six individual mice. The utilization of animals in the investigations was authorized by the Animal Ethics Committee of Noakhali Science and Technology University, under reference No. 61/2021.

### 2.5 Analgesic activity assay

#### 2.5.1 Writhing test

For the acetic acid-induced writhing test, the previously described procedure was applied with certain modifications (Siegmund et al., 1957). A total of twenty mice were divided into four groups of five mice each. Normal saline was administered orally to the control group at a dosage of 10 mL/kg body weight (BW). Aspirin (100 mg/kg BW) was administered to the standard group. The two remaining groups were administered ZrlME at dosages of 200 and 400 mg/kg, respectively. After one-hour therapy, all the experimental animals received an intraperitoneal injection of acetic acid (0.2 mL, 3%) to produce painful writhing. In order to calculate the writhing movements, they were granted entry to an observation chamber, whereby the act of observation transpired during a duration ranging from 5 to 15 min. We defined writhing as trunk twisting, abdominal contractions, body lengthening, and/or the pelvis terminating with the stretching of the limbs. The data was processed using the mean percent inhibition of writhing (PIW):

$$\text{PIW}=\frac{\text{Count}\;\text{of}\;\text{writhes}\;\left(\text{control}\right)\;–\;\text{Count}\;\text{of}\;\text{writhes}\;\left(\text{treated}\right)}{\text{Count}\;\text{of}\;\text{writhes}\;\left(\text{control}\right)}\times 100$$

#### 2.5.2 Formalin-induced nociception model

The formalin-induced licking test was conducted with slight adjustments to the procedure previously outlined in the literature (Hunskaar and Hole, 1987). The control group received a dosage of 10 mL/kg body weight of normal saline, whereas the experimental group was administered a dosage of 100 mg/kg body weight of aspirin. The ZrlME was provided to two groups at doses of 200 and 400 mg/kg. Following an hour of individual treatments, all test animals received a subcutaneous injection of 20 µL of a 1% formalin solution with the intention of inducing discomfort. Subsequently, the mice were confined within a designated space for the purpose of conducting observations, wherein any instances of bites or licks directed towards the injected paw were duly noted and considered indicative of distress. The neurogenic or initial response time was observed to range from 0 to 5 min, but the late or inflammatory pain response was documented to occur between 20 and 30 min following the injection. The data were subjected to analysis utilizing the subsequent equation, which represented the mean percentage inhibition of the licking reaction (PIL):

$$\text{PIL}=\frac{\text{Licking}\;\text{reaction}\;\text{time}\;\left(\text{control}\right)\;–\;\text{Licking}\;\text{reaction}\;\text{time}\;\left(\text{treated}\right)}{\text{Licking}\;\text{reaction}\;\text{time}\;\left(\text{control}\right)}\times 100$$

#### 2.5.3 Hot plate test

The hot plate test was conducted using an analgesiometer, following the methodology outlined by Yi et al. (2010) (Yi et al., 2010). During the course of the experiment, the temperature of the plate was maintained at a constant value of 131 ± 1.6.9°F. The mice that exhibited a response time of less than or equal to 10 s to the heat stimulus on the plate were selected for the future phase of the experiment. The control group received a dosage of normal saline at a rate of 10 mL per kilogram of body weight, while the standard group received a dose of Ketorolac at a rate of 10 mg per kilogram of body weight. The remaining two experimental groups were administered doses of 200 mg/kg and 400 mg/kg of extract. The latency duration was thereafter assessed at designated time intervals, and the termination of the latency phase was defined as the point at which the mouse initiated jumping or paw licking. In order to mitigate the risk of tissue damage, a predetermined time limit of 20 s was established. The data was subjected to analysis mean percent maximal effect (%MPE) utilizing the subsequent equation:

%MPE = [(La-Lb)/(C-Lb)] × 100; Where, La represents the latency observed after administering a drug, Lb represents the latency observed before administering the drug, and C indicates the cut-off time.

### 2.6 Anti-inflammatory activity

Using a paw edema test with carrageenan, anti-inflammatory activity was assessed. Animals were initially divided into four groups of five animals each. A Vernier scale was used to measure each rat’s right hind paw size. Then, we administered oral doses of 10 mL/kg BW of normal saline for the control group and 10 mg/kg BW of indomethacin for the standard group, respectively. The remaining two test groups received 200 mg/kg and 400 mg/kg doses of ZrlME. After the above agents were administered for 30 min, 0.1 mL of 1% carrageenan was injected into the plantar tissue of the right hind paw of each rat to cause edema (Vázquez et al., 1996). Paw thickness was assessed at “0 h,” right before the carrageenan injection, and then at “1, 2, 3, and 4 h” (hours) later. The increase in paw thickness was calculated as the difference between paw thickness at “0 h” and paw thickness at the relevant hours.

### 2.7 Antipyretic activity

A well-established methodology was employed to assess the antipyretic (Reza et al., 2021). To induce fever, mice were subcutaneously injected with a 30% yeast solution. Following a period of 18 h of fasting, the presence of elevated body temperature in mice, indicative of pyrexia, was ascertained through a measured rise of 1.5°F in rectal temperature. The participants in the placebo group ingested a volume of 10 mL per kilogram of body weight of normal saline. The control group was given a dosage of 150 mg/kg body weight of paracetamol, while the other two groups received dosages of 200 and 400 mg/kg body weight of leaf extract, respectively. The temperature was recorded at intervals of 30 min, 1 h, 2 h, 3 h, and 24 h.

### 2.8 Bioactivity prediction

The bioactivity profile of identified polyphenols, namely 3, 4- dihydroxy benzoic acid, rosmarinic acid, p-coumaric acid, (-) epicatechin, rutin hydrate, trans-ferulic acid, and myricetin, were investigated using PASS program. Pa values (probability “to be active”) and Pi values (probability “to be inactive”) are used to represent predicted data for each molecule for each activity. In the present study, a Pa value beyond 0.7 (pa >0.7) signifies a likely activity level surpassing 70%. Experimental techniques have a higher probability of uncovering a specific pharmacological action when the value of Pa exceeds Pi (Pa > Pi), particularly when the Pa value surpasses 0.7 (Marwaha et al., 2007).

### 2.9 *In silico* studies

#### 2.9.1 Molecular docking

The molecular docking experiments were conducted on the seven discovered ligands in order to assess their potential as *in vivo* inhibitors of cyclooxygenase-2. The 3D crystal structures of proteins were obtained by utilizing the PDB files from the Protein Data Bank (Rose et al., 2016). The selected protein is identified by the accession code 3LN1, also known as COX-2. Prior to utilization, the Discovery Studio 2020 client removed water molecules, other cofactors, and associated ligands (Emon et al., 2020). The 3-dimensional (3D) structures of seven polyphenols and celecoxib, a widely used cyclooxygenase-2 inhibitor, were obtained in SDF format from the PubChem chemical molecule database (Kim et al., 2016). Subsequently, with the Open Babel graphical user interface (GUI), namely version 3.1.1, the ligands and proteins were translated into the PDBQT format (O'Boyle et al., 2011). The protein docking simulation was conducted using AutoDock Tools version 1.5.6 (Huey et al., 2012). Finally, the binding modes were viewed using the Discovery studio 2020 client (Neugebauer et al., 2002).

#### 2.9.2 Molecular dynamics simulation (MDS)

Molecular dynamics simulation (10 ns) was performed for both apo-COX-2 and ligand-bound COX-2. The dynamics simulation was performed using the GROMACS 2021.3 simulation software. The topology for the protein and ligand were generated using the CHARMM36 force field (Omar, 2020). Subsequently, both topologies were merged to create the complex. The protein-ligand system was immersed in water using the TIP3P water model. To nullify the arrangement, counterions were introduced into the system. The entire system underwent energy minimization through 5000 iterations of steepest descent minimization. A position restraint topology was created to impose restraints on the ligands. The ligands were combined with the protein to facilitate temperature coupling. Subsequently, the system was stabilized using NVT and NPT equilibration, and then subjected to MDS. Following the completion of the molecular dynamics simulation, several parameters were assessed root-mean-square deviation (RMSD), radius of gyration, root-mean-square fluctuation (RMSF), and hydrogen bonds number between the protein and ligand.

### 2.10 ADMET prediction

The rate of success in drug discovery and development has significantly increased because to the use of computational screening tools for the examination of prospective metabolites’ absorption, distribution, metabolism, excretion, and toxicity (ADMET) properties. These analyses of pharmacokinetics provide a descriptive concept of the action of the target metabolites in the body of human and forecast their potential as therapeutic candidates. Human intestine absorption, plasma protein binding (PPB), Caco-2 cell permeability, blood-brain barrier penetration, skin permeability, Madin-Darby Canine Kidney (MDCK) cell permeability, and toxicity features like a mutagenic or irritating effect are examples of pharmacokinetic properties. The blood-brain barrier (BBB) penetration is a crucial aspect of drug distribution for therapeutic prospects in the central nervous system (CNS). CNS active refers to drug candidates that have the best BBB penetration potential. It must be greater than 0.40 (>0.40) for their BBB penetration rate. Greater than 90% plasma protein binding indicates a drug candidate that is strongly bound, whereas a percentage of less than 90% indicates a candidate that is weakly bound. Additionally, *in vitro* models for Caco-2 and MDCK cells’ permeability have been successfully applied to predict oral drug candidates’ intestine absorption. Metabolites are classified as poorly, moderately, or highly permeable based on their permeability through Caco-2 cells. A metabolite is highly permeable via Caco-2 cells if its value is greater than 70, moderately permeable through Caco-2 cells if its value is between 4 and 70, and weakly permeable through Caco-2 cells if its value is less than 4. Likewise, a score for MDCK cell permeability >500 indicates that the metabolite is highly permeable, a value between 25 and 500 indicates that it is moderately permeable, and values less than 25 indicate that it is weakly permeable. Drug candidates appropriate for oral delivery can also be found through studies on human intestinal absorption (HIA) and skin permeability. Considering their increasing negative value, substances in an *in silico* investigation exhibit features like skin permeability. The percentage of human intestinal absorption (%HIA) is used to calculate the bioavailability of drugs administered via the hepatic portal vein; a number between 70 and 100 indicates good absorbance. A therapeutic candidate with promising pharmacological characteristics may be eliminated due to toxicity concerns throughout the drug design process. A chemical is supported as a safe medication candidate when the *in silico* toxicity results are negative.

### 2.11 Risk assessment for bioactivity and toxicity

Using molinspiration and Osiris property explorer, seven chosen ligands were assessed for a number of bioactivities and toxicity risks. Nuclear receptor-ligand (NRL), G protein-coupled receptor ligand (GPCRL), kinase inhibitor (KI), ion channel modulator (ICM), enzyme inhibitor (EI), and protease inhibitor (PI) interaction were among the qualities expected for the bioactivity evaluation. Factors including drug score and drug likeness were used to estimate toxicity risks. The predicted analysis indicated that the chosen ligands were nontoxic substances.

### 2.12 Statistical analysis

The statistical studies were performed using GraphPad Prism software, specifically version 8.0.2. The data was subjected to statistical analysis using a one-way analysis of variance (ANOVA) followed by the Bonferroni *post hoc* test. A significance level of *p* < 0.05 (*) was used to determine statistical significance.

## 3 Results

### 3.1 Phenolic composition

**FIGURE 1:**
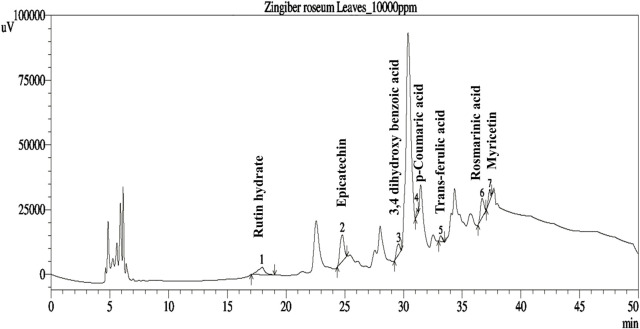
The polyphenols and their respective peaks identified in the HPLC-DAD analysis.

The phenolic profiles of ZrlME are presented in Table 1 and Figure 1. The extract included seven polyphenolic metabolites that were identified by comparing their retention times of standard. (-) Epicatechin (108.98 mg/100 g) was found to be the most prevalent in leaf extracts. The leaves extract also included significant amounts of rosmarinic acid (35.26 mg/100 g), rutin hydrate (17.37 mg/100 g), and 3,4 dihydroxy benzoic acid (12.06 mg/100 g), whereas the least amounts of myricetin (7.89 mg/100 g), trans-ferulic acid (6.24 mg/100 g), and p-Coumaric acid (2.10 mg/100 g) were identified.

**TABLE 1:** Compounds identified in *Zingiber roseum* leaves extract.

Name of standard	Content (mg/100 g DE)
Gallic acid	Nd.
Rutin hydrate	17.37 ± 0.43
Catechin hydrate	Nd.
Catechol	Nd.
(-) Epicatechin	108.98 ± 1.47
Syringic acid	Nd.
3,4 dihydroxy benzoic acid	12.06 ± 0.28
p-Coumaric acid	2.10 ± 0.55
Caffeic acid	Nd.
Trans-Ferulic acid	6.24 ± 0.33
Vanillic acid	Nd.
Rosmarinic acid	35.26 ± 0.20
Myricetin	7.89 ± 0.24
Quercetin	Nd.
Trans-Cinnamic acid	Nd.
Kaempferol	Nd.

DE, Dry extract and Nd., Not detected

### 3.2 Analgesic activity

#### 3.2.1 Writhing test

Control mice writhed 31.6 times on average after being treated with acetic acid. A dose-dependent inhibition of writhes was observed in mice treated with leaf extract (Table 2). Both dosages of ZrlME prevented writhing by 22.78% and 58.28%, respectively, in comparison to the control group. On the other hand, the aspirin treated group decreased wreathing by 68.35% as compared to the control group.

**TABLE 2:** The impact of *Z. roseum* (leaf) extract on the suppression of acetic acid-induced writhing.

Group	Counting writhing	% of inhibition
Control	31.50 ± 4.31	**-**
Aspirin	10.00 ± 1.14**	68.35
ZrlME 200 mg/kg	24.40 ± 2.11	22.78
ZrlME 400 mg/kg	13.20 ± 1.83**	58.28

The Mean ± SEM (*n* = 5) is used to represent all values; ZrlME represents methanol extract of *Z. roseum*. In comparison to control, *p < 0.05 and **p < 0.01, are deemed significant.

#### 3.2.2 Formalin induced paw-licking test

**FIGURE 2:**
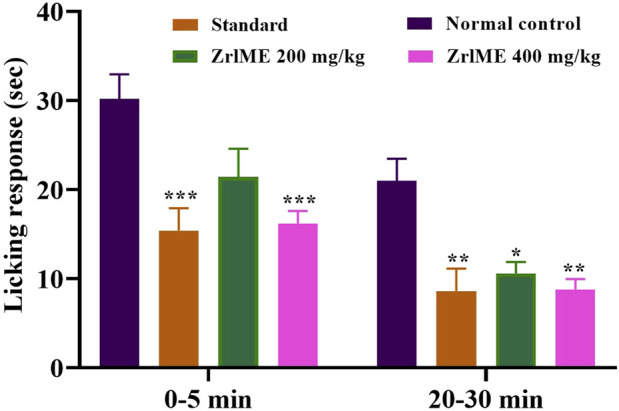
Results of *Z. roseum* leaves methanol extract (ZrlME) and standard (aspirin) in formalin-induced test. Values are stated as mean ± SEM, (*n* = 5); *p < 0.05, **p < 0.01, and ***p < 0.001 are considered significant compared to control.

In the early and late phases of the formalin test, the control group’s average licking time was 30.20 and 21 s, respectively. ZrlME dose-dependently suppressed the licking response during both stages (Figure 2). ZrlME 200 mg/kg and ZrlME 400 mg/kg decreased paw licking by 29.13% and 46.35%, respectively, in the initial phase, which was comparable to standard (49%) (Table 3). However, in the late phase, aspirin’s maximum inhibition was noted (59.04%), and ZrlME 200 mg/kg demonstrated 58.09% inhibition.

**TABLE 3:** Results of *Z. roseum* leaves methanol extract (ZrlME) and standard (aspirin) on % inhibition of the licking reaction.

Group	% of inhibition of licking	
	Early phase (0–5 min)	Late phase (20–30 min)
Aspirin	49.00	59.04
ZrlME 200 mg/kg	29.13	49.52
ZrlME 400 mg/kg	46.35	58.09

#### 3.2.3 Hot plate test

**FIGURE 3:**
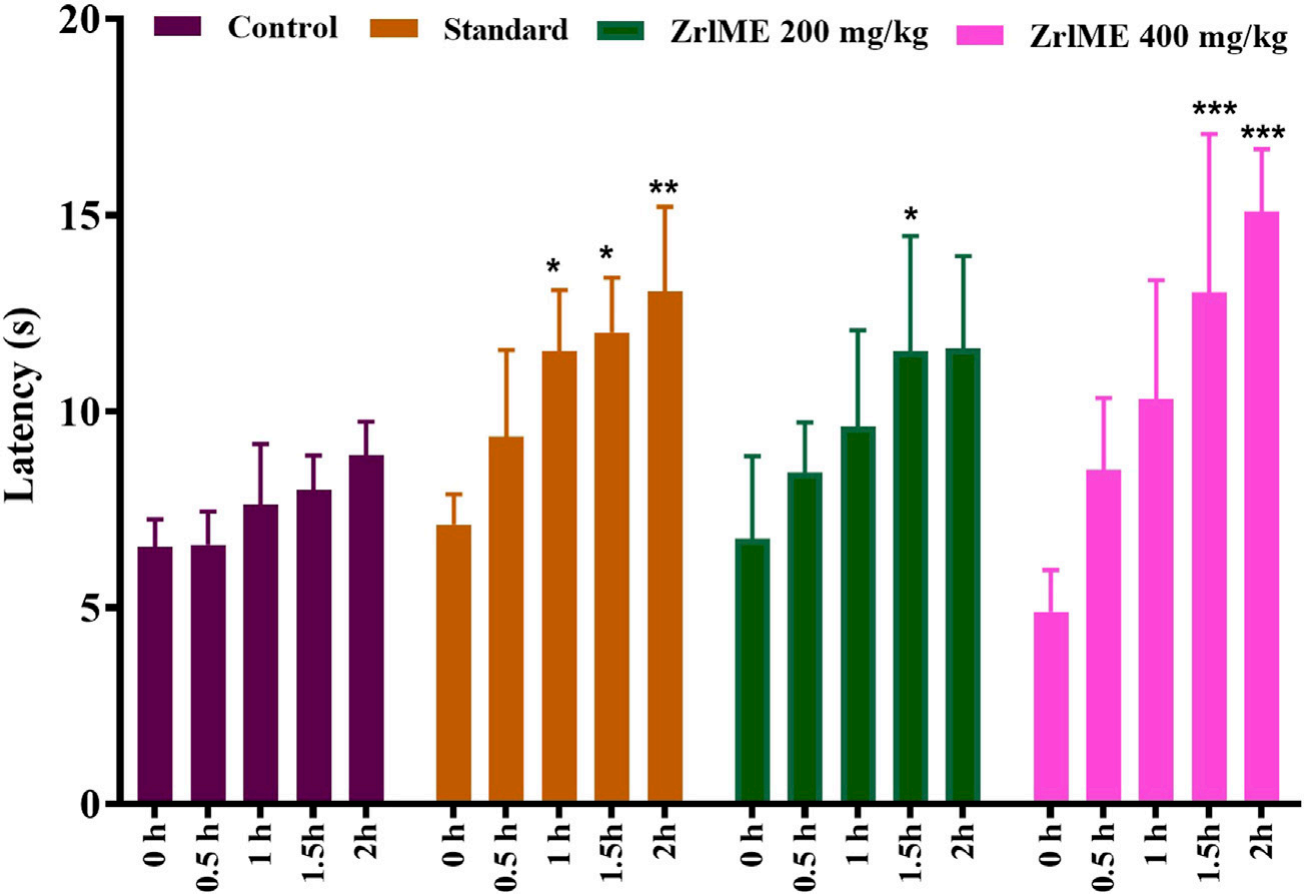
Effect of methanol extract of *Z. roseum* leaves (ZrlME) and standard (ketorolac) on the hot plate test. Values are stated as mean ± SEM, (*n* = 5); Following an ANOVA and Bonferroni test, all data were evaluated. *p < 0.05, **p < 0.01, and ***p < 0.001 are considered significant compared to control.

**FIGURE 4:**
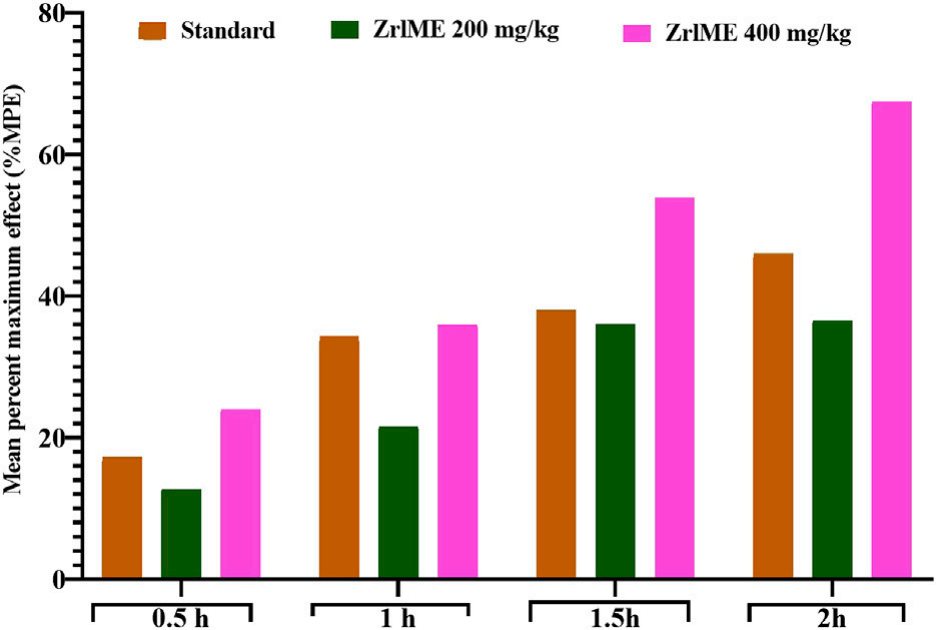
Effect of ZrlME and standard (ketorolac) on the hot plate test. This panel presented the mean percent maximum effect (%MPE) of standard and various dosages of plant extract.

The plant extract demonstrated a dose-dependent increase in latency against thermal stimuli (Figure 3). At 1 hour, antinociceptive activity from ZrlME at 400 mg/kg dosage (53.92%) was found higher than that from standard ketorolac (37.99%). A similar trend was also observed after 2 h, where ZrlME (400 mg/kg) produced a higher mean percent maximal effect (67.46%) than conventional ketorolac (46.03%) (Figure 4).

### 3.3 Anti-inflammatory activity

The results of the anti-inflammatory test are summarized in Table 4. After 1 h of the injection of carrageenan, notably, a significant reduction (*p* < 0.001) of edema was detected at both doses of extracts (200 and 400 mg/kg) from ZrlME. At 4 h post-injection of carrageenan, ZrlME (200 and 400 mg/kg) demonstrated higher inhibition (68.20% and 72.73%, respectively) than indomethacin (68.18%). The results as a whole suggested that the extract is effective and has noticeable outcomes.

**TABLE 4:** Anti-inflammatory activity of *Z. roseum* leaves methanol extract (ZrlME) against paw edema test.

Treatment	0 h	1 h	2 h	3 h	4 h
Control	2.36 ± 0.21	3.00 ± 0.10	2.88 ± 0.05	2.84 ± 0.09	2.80 ± 0.12
Std 10 mg/kg	2.38 ± 0.13	2.76 ± 0.06** (40.63%)	2.64 ± 0.04** (50.00%)	2.60 ± 0.12** (54.16%)	2.52 ± 0.08*** (68.18%)
ZrlME 200 mg/kg	2.28 ± 0.08	2.68 ± 0.08*** (37.5%)	2.56 ± 0.09*** (46.15%)	2.48 ± 0.13*** (58.33%)	2.42 ± 0.16*** (68.20%)
ZrlME 400 mg/kg	2.28 ± 0.08	2.59 ± 0.11*** (51.56%)	2.48 ± 0.11*** (61.54%)	2.44 ± 0.08*** (66.67%)	2.40 ± 0.07*** (72.73%)

Values are stated as mean ± SEM, (*n* = 5); ZrlME represents methanol extract of *Z. roseum*; Std represents the reference standard, indomethacin. In comparison to control, *p < 0.05, **p < 0.01, and ***p < 0.001 are deemed significant.

### 3.4 Antipyretic activity

Rectal temperatures in all animals boosted after 18 h of yeast injection, as demonstrated in (Table 5). Following treatment with the ZrlME, the elevated rectal temperature was decreased in a dose-dependent manner. The antipyretic effect began immediately following the extract treatment. After 0.5, 1, 2, 3, and 24 h of therapy, the temperature was considerably lowered by 1.08°F, 1.43°F, 1.83°F, 2.23°F, and 3.17°F for ZrlME at a dose of 200 mg/kg, and by 1.44°F, 2.14°F, 3.00°F, 3.15°F, and 3.97°F for ZrlME at a dose of 400 mg/kg. Standard paracetamol at 150 mg/kg resulted in a maximum temperature drop of 4.62°F after 24 h, while ZrlME 200 mg/kg and ZrlME 400 mg/kg resulted in maximum temperature reductions of 3.17°F and 3.97°F, respectively.

**TABLE 5:** Antipyretic effect of *Z. roseum* leaves methanol extract (ZrlME)) on Baker’s yeast test.

Group	Rectal temperature (°F)^a^	Rectal temperature measured following the appropriate treatment (°F)				
		0.5 h	1 h	0.5 h	3 h	0.5 h
Control	101.72 ± 0.26	101.56 ± 0.34	101.72 ± 0.26	101.56 ± 0.34	101.72 ± 0.26	101.56 ± 0.34
Paracetamol	101.4 ± 0.60	99.3 ± 1.36	101.3 ± 0.70	99.3 ± 1.27	101.3 ± 0.70	99.3 ± 1.27
ZrlME 200 mg/kg	99.64 ± 0.52	98.56 ± 0.28*	99.64 ± 0.52	98.56 ± 0.28*	99.64 ± 0.52	98.56 ± 0.28*
ZrlME 400 mg/kg	99.80 ± 0.46	98.36 ± 0.47*	99.80 ± 0.46	98.36 ± 0.47*	99.80 ± 0.46	98.36 ± 0.47*

^a^ Rectal temperature after 18 h of yeast injection Values are stated as mean ± SEM, (n = 5); ZrlME represents methanol extract of *Z. roseum*.

In comparison to control, *p < 0.05, **p < 0.01, and ***p < 0.001 are deemed significant.

### 3.5 Predicting biological activity

On the basis of the Pa value (Pa >0.07), the bioactivity of each polyphenol was selected. The predicted biological characteristics and potential MOA for these substances are shown in Supplementary Table S1.

### 3.6 *In-silico* study

#### 3.6.1 Molecular docking

**FIGURE 5:**
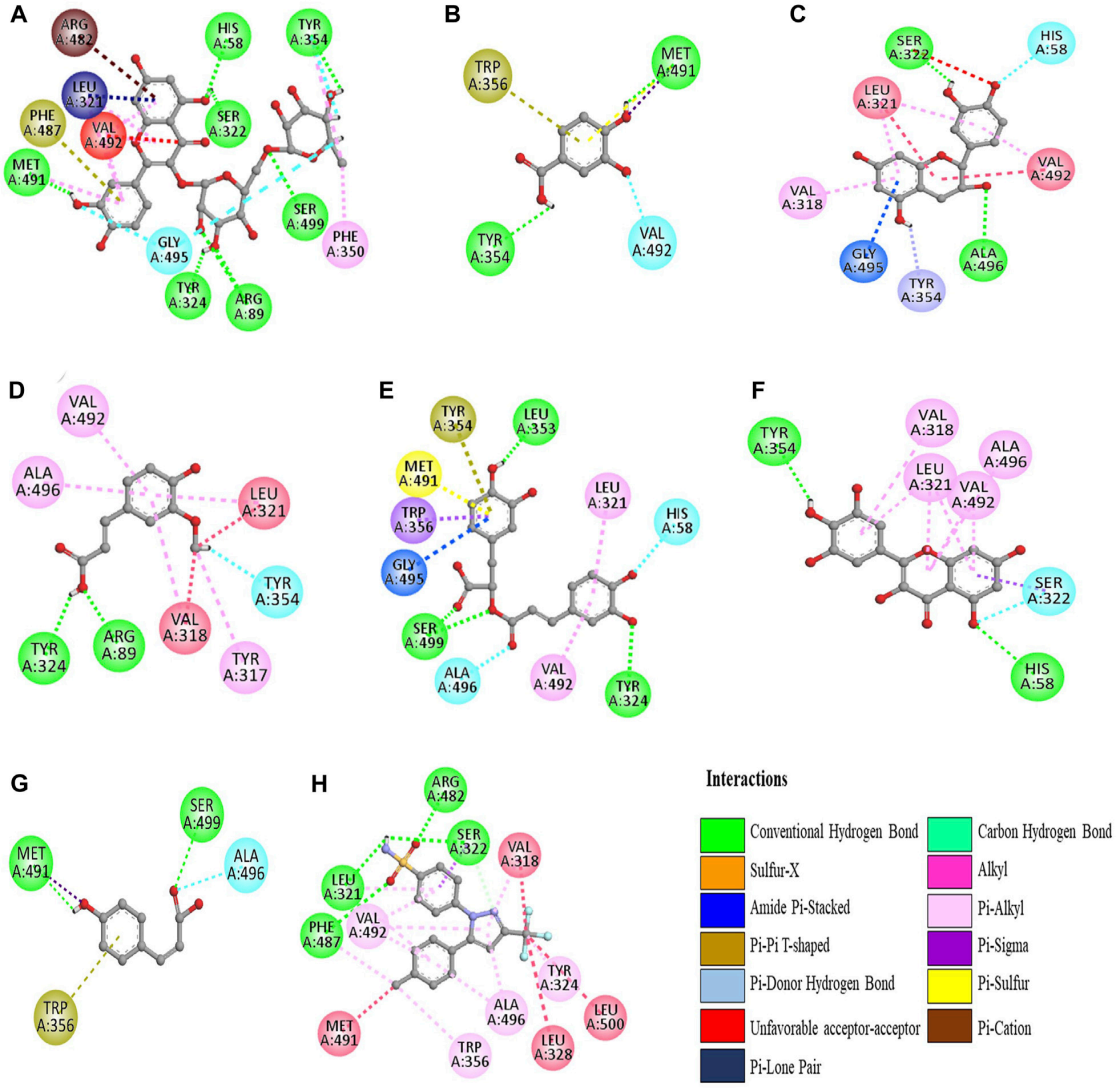
2D interactions of identified polyphenols, **(A)** rutin hydrate, **(D)** trans-ferulic acid, **(G)** p-coumaric acid, **(B)** 3,4 dihydroxy benzoic acid, **(E)** rosmarinic acid, **(H)** celecoxib, **(C)** epicatechin, **(F)** myricetin, with protein COX-2 (PDB ID:3LN1) (AutoDock Vina anticipated this position).

**FIGURE 6:**
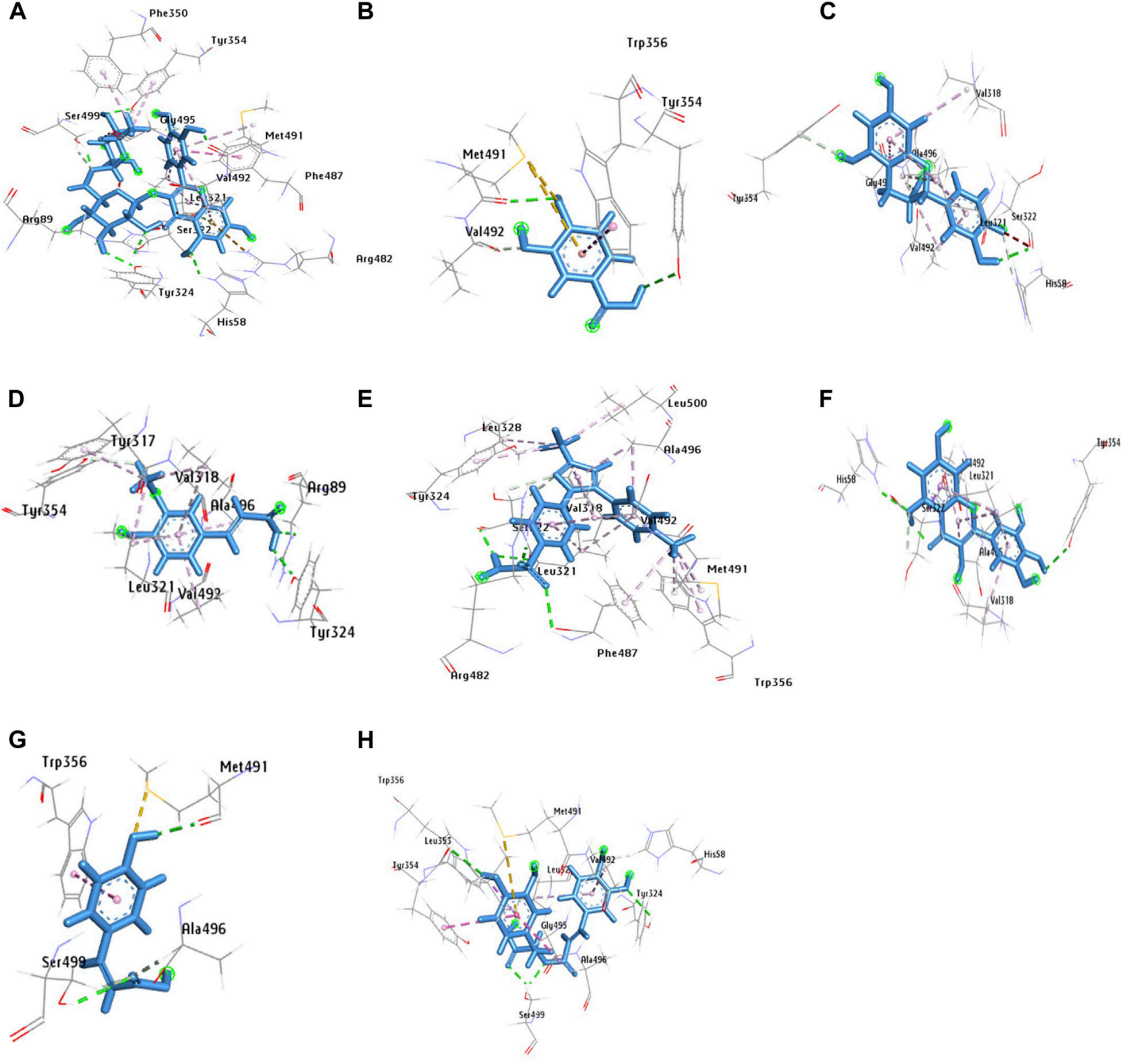
3D configuration of identified polyphenols, **(A)** rutin hydrate, **(D)** trans-ferulic acid, **(G)** p-coumaric acid, **(B)** 3,4 dihydroxy benzoic acid, **(E)** rosmarinic acid, **(H)** celecoxib, **(C)** epicatechin, **(F)** myricetin, with protein COX-2 (PDB ID:3LN1) (AutoDock Vina anticipated this position).

The molecular docking was conducted to examine the interaction between the phytocompounds identified in ZrlME and COX-2. The findings of this analysis are presented in Supplementary Table S2. The 2D and 3D interactions between polyphenols and the cyclooxygenase-2 enzyme are depicted in Figures 5, 6, respectively. Seven polyphenols that target particular receptors had their free binding energies compared to celecoxib, a powerful COX-2 inhibitor, and non-steroidal anti-inflammatory medication. Rutin hydrate had a free binding energy with COX-2 of −8.5 Kcal/mol, which was nearly identical to standard celecoxib’s free binding energy of −8.7 Kcal/mol. Also, the significant free binding energy of myricetin was −7.7 Kcal/mol and that of epicatechin was −7.0 Kcal/mol.

#### 3.6.2 Molecular dynamics simulation (MDS)

**FIGURE 7:**
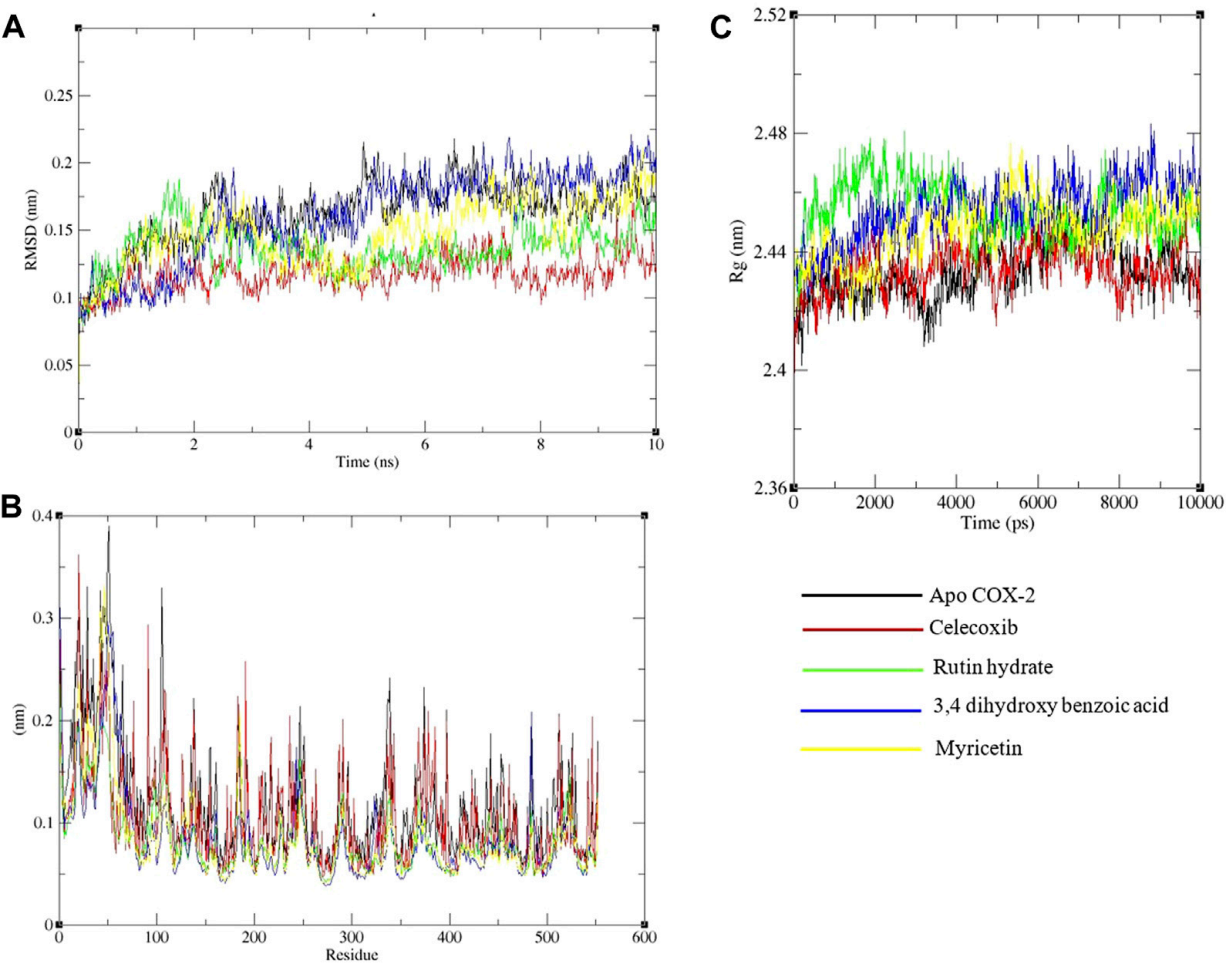
Analysis of a 10 ns molecular dynamics simulation of Cox-2 with ligands. **(A)** Root mean square deviation (RMSD), **(B)** Root mean square fluctuations on a residue-wise basis, and **(C)** Radius of gyration.

A 10 ns duration simulation was conducted to study the dynamics of the conventional medicine celecoxib and polyphenols with COX-2 enzyme, as shown in Figure 7. MDS has been performed for the apo form of COX-2. The root mean square deviation (RMSD) of the alpha-carbon of apo COX-2 remained between 0.1 nm and 0.25 nm throughout the simulation, suggesting a higher level of stability. When celecoxib is coupled to COX-2, the RMSDs for the alpha carbon range from 0.1 to 0.15 nm, suggesting a high level of structural stability. Rutin hydrate, 3,4 dihydroxy benzoic acid and myricetin exhibited comparable RMSD values to celecoxib (Figure 7A). Based on the data presented in Figure 7B, the RMSF values for both the apo and ligand-bound forms of COX-2 are nearly identical. Upon examining the hydrogen bonding area of COX-2 and ligands, it becomes apparent that the residues involved in hydrogen bonding with the ligands demonstrated less variability compared to apo COX-2.

**FIGURE 8:**
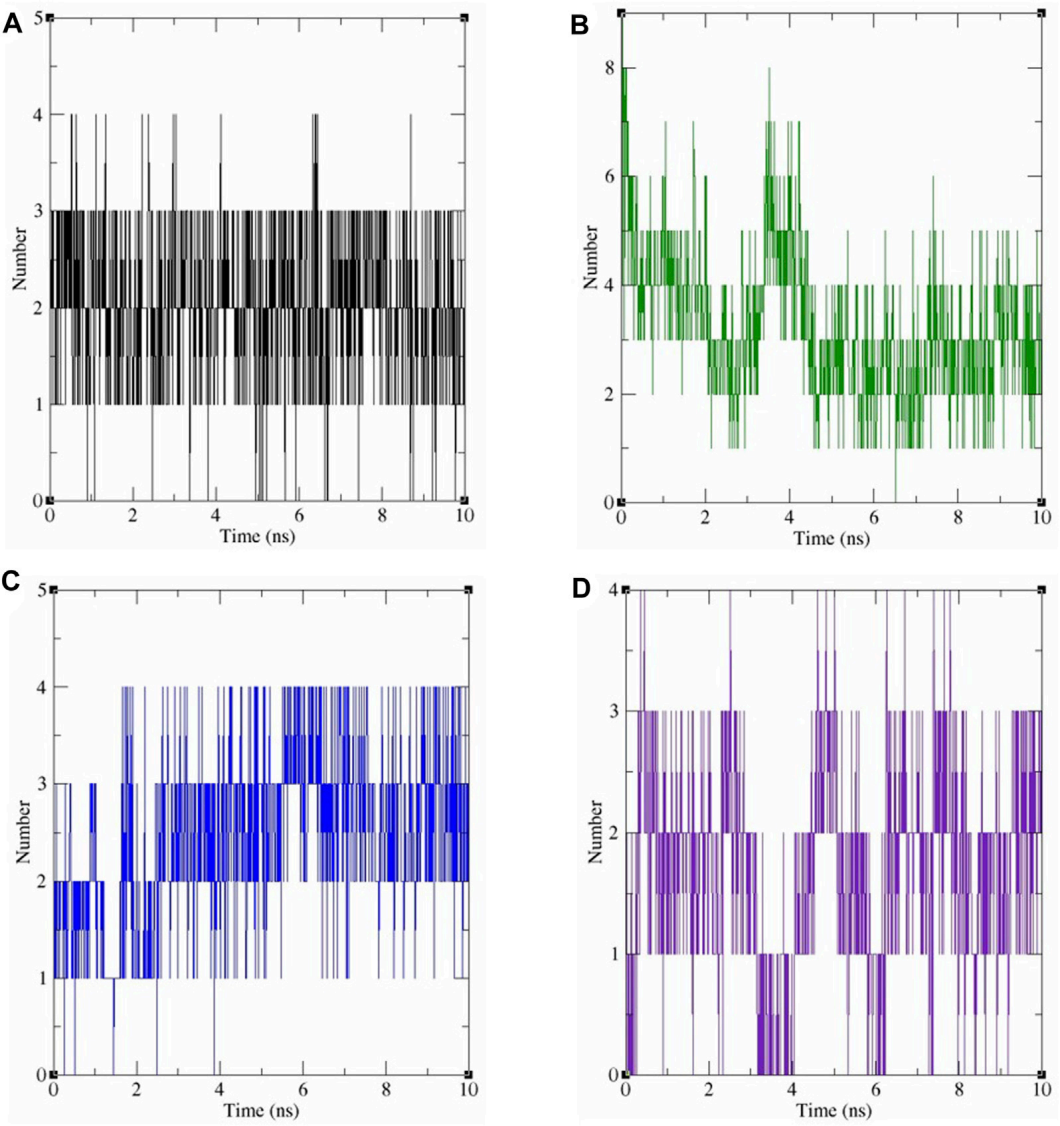
The number of hydrogen bonds produced during 10 ns dynamics simulation between COX-2 and the following ligands: **(A)** celecoxib, **(B)** rutin hydrate, **(C)** 3,4 dihydroxy benzoic acid, and **(D)** myricetin.

The protein’s structural compactness is measured by the radius of gyration (Rg). The more compact the protein, the smaller the variations in the Rg. The consistent uniformity of the Rg (Figure 7C) throughout the simulation indicates improved system rigidity. Ligand and protein stability are highly dependent on hydrogen bond interactions [36]. The observed intermolecular hydrogen bonds formed by ligands and COX-2 during the 10 ns MDS are displayed in Figure 8. Throughout the 10 ns duration, COX-2 and the control drug celecoxib consistently formed 1-3 hydrogen bonds (Figure 8A). At the beginning of the dynamics, rutin hydrate and COX–2 form 4–7 hydrogen bonds; however, by the end, this number decreases to 2–5 (Figure 8B). 3,4 Dihydroxy benzoic acid produced 2-4 hydrogen bonds throughout the simulation (Figure 8C). It's noteworthy that both of these polyphenols exhibited a higher frequency of hydrogen bond formation compared to celecoxib when interacting with COX-2. As shown in Figure 8D, myricetin consistently formed 1 to 3 hydrogen bonds during the MDS, mirroring the behavior of celecoxib.

### 3.7 ADMET properties

The investigation of ADMET characteristics (Supplementary Table S3) predicted that all of the chosen ligands have BBB penetration rates in the range of 0.028625–0.758419, which suggests that they are more effective at reaching the central nervous system. Except for rutin hydrate (0.028252), all of the selected ligands had a greater BBB penetration rate compared to the celecoxib (0.027635) standard. Seven ligands were found, with values for *in vitro* Caco-2 cell permeability ranging from 0.656962 to 21.1177 nm/sec, compared to a value of 0.499443 for the reference drug celecoxib. Caco-2 cells had a moderate permeability to 3,4-dihydroxy benzoic acid, trans-ferulic acid, rutin hydrate, rosmarinic acid, and p-coumaric acid but not to other ligands. Epicatechin (100%) and myricetin (96.78%) had significantly higher affinity to plasma protein than celecoxib (91.077216). The percentage of HIA was determined to be between 0.941542 and 92.095876. 3,4 Dihydroxy benzoic acid (74.749630), Trans-ferulic acid (90.603297), and p-Coumaric acid (92.095876) have shown a good absorbance. All the ligands tested negative for skin permeability (−4.5272 to −1.70767). The result of toxicity was also negative, recommending that all selected ligands are safe and non-toxic.

### 3.8 Bioactivity & toxicity risk studies

Supplementary Table S4 provides a summary of seven ligands’ bioactivity and toxicity risk profiles. The results showed that all of the ligands were approximately comparable to the bioactivity properties of the standard celecoxib value. Moreover, the drug-likeness of all the ligands were within range of −2.07–3.31, which was higher than the standard celecoxib value of −8.11. Furthermore, the drug scores of these ligands were estimated to be in the 0.19–0.89 range, which is comparable to the drug scores of celecoxib (0.37). Finally, the study of bioactivity and toxicity reveals that all ligands are safe as potential medicinal agents.

## 4 Discussion

Isolating new therapeutic metabolites from medicinal plants has become increasingly important in recent years. Unfortunately, most of the plants are yet unexplored in terms of their pharmacological and toxicological characteristics, as well as the identification of valuable bioactive metabolites. *Zingiber roseum* (Roxb.) Roscoe is a medicinal plant with undisclosed medicinal and pharmacological effects. Seven polyphenols (3, 4- dihydroxy benzoic acid, (-) epicatechin, rutin hydrate, p-coumaric acid, trans-ferulic acid, rosmarinic acid and myricetin) were identified by HPLC analysis in ZrLME. These plant metabolites have been reported to possess diverse pharmacological actions such as hepatoprotective, anti-arthritic, immunomodulatory, analgesic, anti-arthritic, antipyretic, antibacterial, and anti-inflammatory properties (Benavente-García et al., 1997; Middleton et al., 2000; Puupponen-Pimiä et al., 2001; Manach et al., 2005b).

Acetic acid is the causative agent for the augmentation of capillary permeability. Pain is caused by the stimulation of nerve endings, which is activated by the production of different substances that promote pain, such as prostaglandin, peritoneal mast cells and others (Bueno and Fioramonti, 2002). Conventional nonsteroidal anti-inflammatory drugs (NSAIDs) alleviate pain and inflammation by blocking the function of COX in peripheral tissues and primary afferent nociceptors’ signal transduction (Burian et al., 2005). ZrlME 400 mg/kg dose substantially (*p* < 0.001) reduced the writhing responses in our investigation, which could be attributed to the disruption of pain and inflammatory pathways. However, due to the lack of specificity in this investigation, it is imperative to employ a different experimental approach to confirm the results obtained from the writhing test. Therefore, we utilized a biphasic paw licking experiment as a more accurate approach to evaluate the effectiveness of biphasic pain relief. The biphasic nociceptive response is triggered by injecting formalin into the skin, and the response during the first phase (0–5 min) is believed to be caused by the activation of peripheral nociceptors by formalin, leading to the sudden hindrance of activity. Conversely, during the second phase (lasting from 10 to 45 min), the inflammatory response is believed to occur as a result of inflammatory cytokines, such as prostaglandins, serotonin, bradykinin, etc., into the peripheral nociceptors of the spinal cord (Pethő and Reeh, 2012). Since ZrlME 400 mg/kg significantly (*p* < 0.001) inhibited the paw-licking responses in both stages, it is reasonable to infer that there is a potential for suppressing cytokines generated by inflammation in the peripheral or spinal cord.

The hot plate approach demonstrates that various dosages of ZrlME result in a dose-dependent escalation in reaction time, with the most significant inhibition occurring after 2 h of the trial. The opioid μ receptor plays a key role in regulating thermal pain in response to thermal stimuli, and activating this receptor leads to the production of analgesic activity in the spinal cord (Sun et al., 2019). The rise in response time may be centrally regulated as a result of its impact on the opioid receptor. Polyphenols have been found to produce an analgesic effect through the activation of opioid receptors (De Feo et al., 2020). Fields, H. L. (1984) reported that centrally-acting analgesic medications stimulate the release of endogenous peptides through the periaqueductal grey matter, resulting in the suppression of pain signal transmission in the dorsal horn (Fields, 1984). ZrlME also showed the ability to suppress nociceptive response as evaluated using the hot plate test, indicating its promise as a centrally mediated pain reliever.

Carrageenan is commonly employed as a proinflammatory substance in scientific studies to investigate the anti-inflammatory properties of natural substances (Morris, 2003). Injecting carrageenan subcutaneously causes paw swelling or edema. It is well accepted that the induction of edema with carrageenan injection follows a two-phase pattern. The initial phase of the inflammatory reaction, occurring approximately 1–2 h after the injection of carrageenan, often entails the release of bradykinins, histamine, and serotonin from the mast cells in the injured tissue (Myers et al., 2019). In contrast, during the second phase (often occurring 3–6 h after carrageenan injection), the inflammatory response is triggered by the activation of several inflammatory mediators, such as IL-6, IL-10, IL-1β, TNF-α, and different arachidonate metabolites like leukotrienes and prostaglandins (Loram et al., 2007). The ZrlME dose of 400 mg/kg had the most pronounced effect during the fourth hour in our experiment. Therefore, it may be inferred that this dosage of extract exhibited anti-inflammatory effects via impeding the release of inflammatory mediators.

**FIGURE 9:**
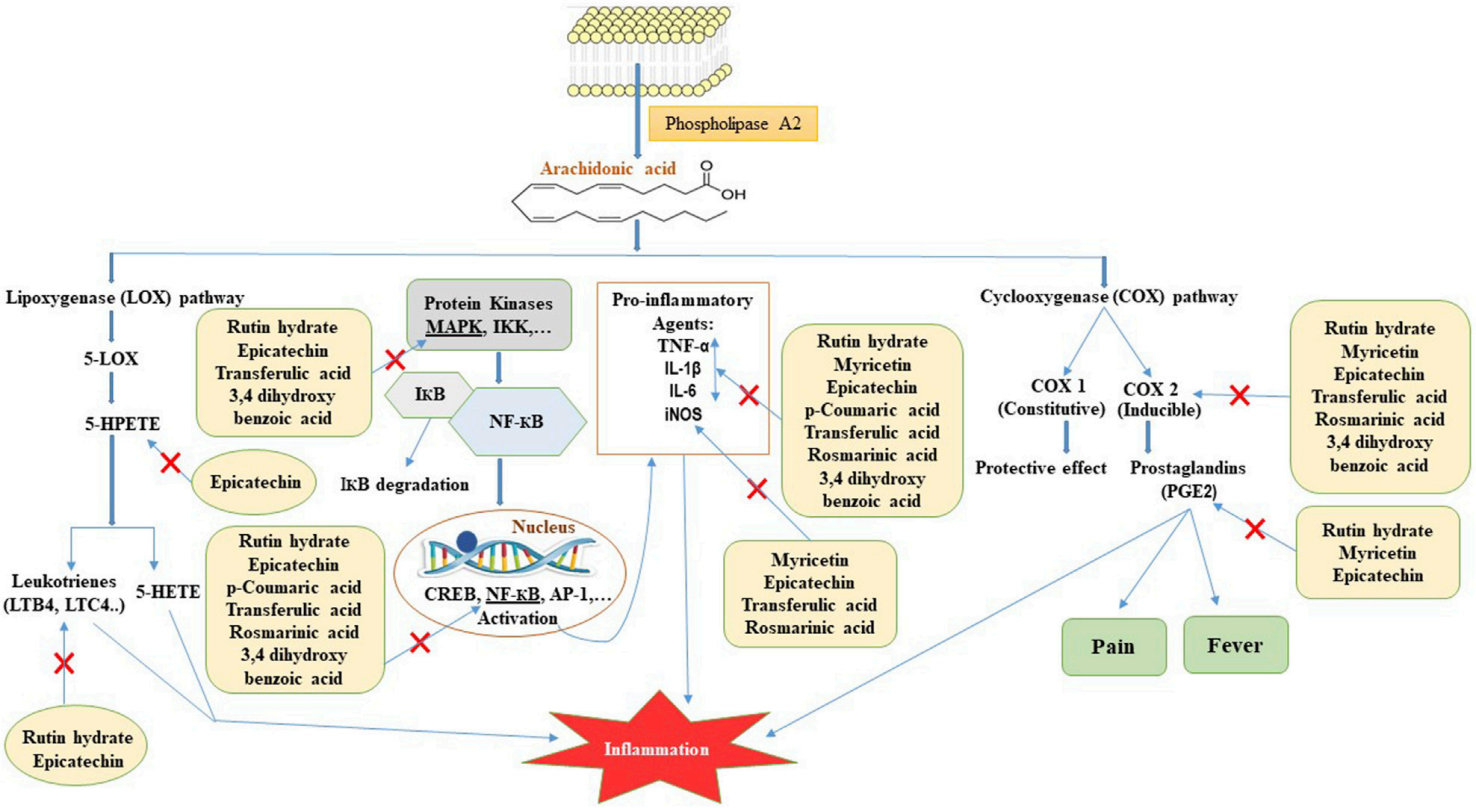
Possible mode of action for polyphenols identified in ZrlME in reducing inflammation. 5-LOX, 5-lipoxygenase; 5-HPETE, 5-hydroperoxyeicosatetraenoic acid; 5-HETE, 5-hydroxyeicosatetraenoic acid; LTB4, leukotriene B4; LTC4, leukotriene C4; COX 1, cyclooxygenase 1; COX 2, cyclooxygenase 2; PGE2, prostaglandin E2; MAPK, mitogen-activated protein kinase; IKK, IКB kinase; IКB, inhibitor of kappa B; NF-КB, nuclear factor kappa B; CREB, cAMP response element-binding protein; AP-1, activator protein-1; TNF-α, tumour necrosis factor-alpha; IL-1β, interleukin-1 beta; IL-6, interleukin-6; iNOS, inducible nitric oxide synthase.

According to previous reports, the injection of carrageenan caused the release of arachidonic acid (AA) and lactate dehydrogenase from pleural cells in rats (Lucetti et al., 2010). AA produces three main categories of eicosanoids: prostaglandins, thromboxanes, and leukotrienes, which play a significant role in causing inflammation. Prior research demonstrated that polyphenols has the ability to impede the COX-2 and LOX-5 enzyme, which plays a crucial role in the synthesis of prostaglandins and leukotrienes from AA (Stoner and Wang, 2013). Hence, the suppression of the eicosanoids signaling pathway could potentially explain the anti-inflammatory impact of ZrlME (Figure 9).

Reactive oxygen species (ROS) induce alterations in inhibitor of nuclear factor kappa B (IκB) proteins, which serve as inhibitors of NF-κB kinase (IKK) (Gloire et al., 2006). This alteration results in the deterioration of IκB, causing the liberation of NF-κB to migrate to the nucleus and stimulate the production of several genes that produce inflammatory proteins, either independently or in conjunction with other transcription factors (Gloire et al., 2006). Hence, suppressing ROS-mediated signaling pathways would be an optimal therapeutic approach for controlling inflammatory diseases. According to earlier reports, rosmarinic acid exhibits anti-inflammatory effects by blocking the NF-κB signaling pathway (Lv et al., 2019). Myricetin is a promising polyphenol that exerts anti-inflammatory effects through many pathways. It has the ability to inhibit the synthesis of multiple pro-inflammatory substances, such as nitric oxide (NO), inducible nitric oxide synthase (iNOS), tumour necrosis factor-alpha (TNF-α), interleukin-6 (IL-6), prostaglandin E2 (PGE2), and interleukin-12 (IL-12) (Figure 9) (Song et al., 2021). Earlier studies also reported that epicatechin has anti-inflammatory properties by decreasing the synthesis of inflammatory substances such as TNF-α, NO, PGE2, IL-1β, IL-6 MAPKs, NF-κB, and JAK2/STAT3 signaling pathways (Figure 9) (Morrison et al., 2014; Amanat et al., 2021). Evidence also showed that rutin can effectively alleviate inflammation by decreasing the levels of pro-inflammatory markers such as TNF-α, IL-6, COX-2, IL-1β (Figure 9) (Hasan et al., 2022; Muvhulawa et al., 2022). The aforementioned polyphenols have been found in significant quantities in ZrlME. Therefore, the remarkable anti-inflammatory activity of ZrlME might be attributed via one of the mentioned pathways.

Fever is an immune system-related acute phase reaction. Fever develops when the hypothalamus’s set point is disrupted (Javed et al., 2020). Fever is caused by simple injury of tissue, which causes inflammation, or by the activity of lipopolysaccharides termed pyrogens, which cause leukocytes to produce cytokines, according to prior study. Prostaglandin E2 (PGE2) is the primary mediator of fever, and it is produced by COX-2 in response to the stimulation of cytokines in the hypothalamus. Microvascular endothelial cells, which abundantly produce COX-2 in response to stress, are the primary cell type in the CNS that create PGE2. The pyretic response is also influenced by activated macrophages, leukocytes, and endothelial cells in inflammatory regions (Taïwe et al., 2011). Subcutaneous administration of the exogenous pyrogen, baker’s yeast, augments the production and secretion of several cytokines such as interleukins (IL-1, IL-6), prostaglandins, TNF, and others (Roy et al., 2019). Most antipyretics work by blocking PGE2 in the hypothalamus to reduce fever, but peripheral active leukocytes and endothelial cells may also be potential therapeutic targets (Javed et al., 2020). At both doses, ZrlME demonstrated a strong antipyretic activity. Both doses, as well as standard paracetamol, resulted in a fast decrease in high rectal temperature, which steadily decreased over time. Paracetamol suppresses prostaglandin synthesis by inhibiting COX pathway; the phytochemical(s) in ZrlME may inhibit COX activity, bringing the body temperature back to normal.

Molecular docking is a popular and widely used method in the realm of drug development. Better understanding of binding mechanisms and the potential for binding of various proteins can be attained using this method (Khan et al., 2019). The seven identified ligands were assessed as possible cyclooxygenase-2 inhibitors utilizing molecular docking and MDS. The phenolic metabolites had high binding affinities (range from 8.5 to 5.8 Kcal/mol) to the COX-2 protein and formed a notable number of hydrogen bonds, surpassing those of celecoxib. The number of hydrogen bonds formed by rutin hydrate, 3,4 dihydroxy benzoic acid and myricetin with COX-2 during the dynamics simulation was comparable to that of celecoxib. The most abundant polyphenols, (-) epicatechin, was reported to have a very stable interaction with COX-2 (Mazumder et al., 2022). Talukder et al. (2022) also revealed the enduring association of rosmarinic acid (second most abundant polyphenols in ZrlME) with the active region of COX-2, signifying noteworthy inhibitory effect of ZrlME against COX-2 (Talukder et al., 2022).

## 5 Conclusion

The current study demonstrated promising analgesic, anti-inflammatory, and antipyretic efficacy of the ZrlME. The molecular docking and MDS analysis also indicated that the phenolic metabolites present in ZrlME exhibited potential inhibition of COX-2 activity. The observed pharmacological effects of ZrlME could be attributed to the presence of these bioactive polyphenols. In-depth clinical research on experimental substances is still needed to confirm their efficacy and safety profile. In conclusion, the ZrlME was proved to be a natural, safe remedy for the treatment of analgesia, inflammation, and pyrexia.

## Data Availability

The original contributions presented in the study are included in the article/Supplementary Material, further inquiries can be directed to the corresponding authors.

## Abbreviations

ZrlME, *Zingiber roseum (Roxb.) Roscoe* leaves methanol extract; ANOVA, Analysis of variance; 5HT, 5-hydroxytryptamine; COX-2, Cyclooxygenase-2; PGE2, Prostaglandin E2; PASS, Prediction of activity spectra for substance; SEM, Standard error of mean; Da, Dalton; nm, nanometer; TNF-α, Tumor necrosis factor alpha; BW, Body weight; kg, kilogram; mg, milligram; IL-1, Interleukin-1; μg, microgram.
