# Supplementary material for: Polyphenols and extracts from Zingiber roseum (Roxb.) Roscoe leaf mitigate pain, inflammation and pyrexia by inhibiting cyclooxygenase-2: an in vivo and in silico studies
Source: Front Pharmacol. 2024 Feb 14;15:1344123. doi: 10.3389/fphar.2024.1344123 (PMC10900100; doi:10.3389/fphar.2024.1344123)
Supplement: Supplementary file 1 [file Table1.docx]

**Supplementary Table S1:** Major chemicals from *Z. roseum* leaf extract predicted to have certain biological activity.

| **Name** | **Predicted biological Activity/MOA** |
| --- | --- |
| 3,4 dihydroxybenzoic acid | Anti-inflammatory  Leukotriene-B4 20-monooxygenase inhibitor  Antiinfective |
|  | Prostaglandin-A1 DELTA-isomerase inhibitor |
|  | Peroxidase inhibitor |
|  | Mucomembranous protector |
|  | Histamine release inhibitor |
|  | Antimutagenic |
|  | Antipyretic |
| Epicatechin | Membrane integrity agonist |
|  | Hepatoprotectant |
|  | Mucomembranous protector |
|  | Antioxidant |
|  | Lipid peroxidase inhibitor |
|  | Cardioprotectant |
|  | Histamine release inhibitor |
| Trans-Ferulic acid | Antiinflammatory |
|  | Mucomembranous protector |
|  | Peroxidase inhibitor |
|  | TNF expression inhibitor |
|  | Antipyretic |
|  | Membrane permeability inhibitor |
|  | Antimutagenic |
| Rosmarinic acid | Reductant |
|  | Antihypoxia |
|  | Inhibition of lipid peroxidase |
|  | Agonist of membrane integrity |
|  | Inhibition of membrane permeability |
|  | TNF expression inhibitor |
|  | Antidiabetic |
| Rutin hydrate | Antiinflammatory  Membrane permeability inhibitor |
|  | Inhibition of lipid peroxidase |
|  | Hepatoprotectant |
|  | Proliferative diseases treatment |
|  | Antioxidant |
|  | Kinase inhibitor |
|  | Antineoplastic |
|  | Cardioprotectant |
| Myricetin | Antiinflammatory  Interleukin 4 antagonist  Cardioprotectant |
|  | Inhibition of peroxidase |
|  | Inhibition of mutagenesis |
|  | Inhibition of kinase |
|  | Antioxidant |
|  | Agonist of apoptosis |
|  | Antihemorrhagic |
| p-Coumaric acid | Protector of mucomembrane  Prostaglandin-E2 9-reductase inhibitor  Antiinflammatory |
|  | Antimutagenic |
|  | Antihypoxic |
|  | Reductant |
|  | TNF expression inhibitor  Peroxidase inhibitor  Antiseptic |

**Supplementary Table S2:** The number of hydrogen bonds and the binding affinity were discovered following molecular docking.

| Ligand | No of hydrogen bond | Binding affinity (kcal/mol) |
| --- | --- | --- |
| Celecoxib | 4 | -8.7 |
| 3,4 Dihydroxy benzoic acid | 2 | -5.8 |
| (-) Epicatechin | 2 | -7.0 |
| Trans-ferulic acid | 2 | -6.5 |
| Rosmarinic acid | 4 | -6.2 |
| Rutin hydrate | 8 | -8.5 |
| Myricetin | 2 | -7.7 |
| p-Coumaric acid | 2 | -6.3 |

**Supplementary Table S3:** ADMET Characteristics of phenolic compounds identified in ZrlME.

| Name | BBB-penetration | MDCK | Caco-2 | HIA | PPB | Skin permeation | Toxicity |
| --- | --- | --- | --- | --- | --- | --- | --- |
| 3,4 dihydroxy benzoic acid | 0.442883 | 23.6995 | 18.3046 | 74.749630 | 27.115512 | -3.31434 | None |
| Epicatechin | 0.394913 | 44.3849 | 0.656962 | 66.707957 | 100.0 | -4.29301 | None |
| Trans-ferulic acid | 0.758419 | 228.559 | 21.1177 | 90.603297 | 50.414225 | -1.87204 | None |
| Rosmarinic acid | 0.104434 | 0.20263 | 20.7246 | 62.487577 | 86.242087 | -3.32759 | None |
| Rutin hydrate | 0.028625 | 0.13668 | 9.08073 | 0.941542 | 67.257863 | -4.08573 | None |
| Myricetin | 0.110308 | 4.88225 | 0.991395 | 40.964049 | 96.784810 | -4.5272 | None |
| p- Coumaric acid | 0.694635 | 75.0598 | 21.1093 | 92.095876 | 63.055072 | -1.70767 | None |

**Supplementary Table S4:** Risks associated with compounds’ bioactivity and toxicity.

| Compounds | Structure | Bioactivity | | | | | | Toxicity Risks | |
| --- | --- | --- | --- | --- | --- | --- | --- | --- | --- |
|  |  | GPCRL | EI | PI | NRL | KI | ICM | Drug Likeness | Drug score |
| 3,4 dihydroxy benzoic acid | 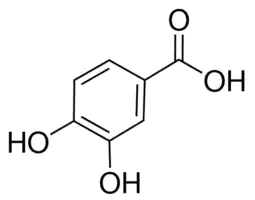 | -0.88 | -0.34 | -1.09 | -0.58 | -1.10 | -0.35 | -0.12 | 0.43 |
| Epicatechin |  | 0.41 | 0.47 | 0.26 | 0.60 | 0.09 | 0.14 | 1.92 | 0.89 |
| Trans ferulic acid |  | -0.47 | -0.12 | -0.81 | -0.14 | -0.72 | -0.30 | 1.12 | 0.19 |
| Rosmarinic acid |  | 0.17 | 0.24 | 0.15 | 0.57 | -0.18 | -0.08 | -2.07 | 0.50 |
| Rutin hydrate | 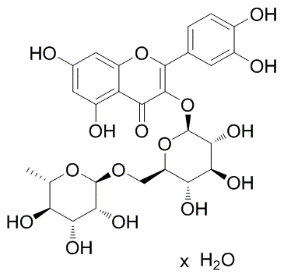 | -0.05 | 0.12 | -0.07 | -0.23 | -0.14 | -0.52 | 3.31 | 0.57 |
| Myricetin |  | -0.06 | 0.30 | -0.20 | 0.32 | 0.28 | -0.18 | 0.75 | 0.47 |
| p-Coumaric acid | 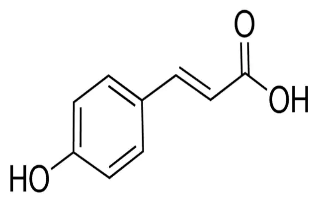 | -0.56 | -0.15 | -0.87 | -0.12 | -0.91 | -0.26 | 0.58 | 0.79 |
| Celecoxib | 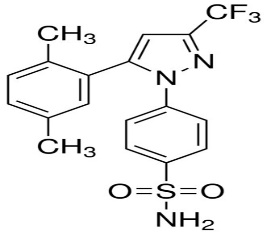 | -0.06 | 0.17 | -0.06 | -0.28 | 0.01 | -0.27 | -8.11 | 0.37 |
|  |  |  |  |  |  |  |  |  |  |
